# Towards a South African model of language-based learning disability

**DOI:** 10.4102/sajcd.v66i1.634

**Published:** 2019-11-22

**Authors:** Xoli Mazibuko, Penelope Flack, Jane Kvalsvig

**Affiliations:** 1 Discipline of Speech Language Pathology, University of KwaZulu-Natal, Durban, South Africa; 2 School of Public Health, University of KwaZulu-Natal, Durban, South Africa

**Keywords:** clinical assessments, early indicators, language-based learning disability, learning disability, systems approach

## Abstract

**Background:**

This conceptual article is inspired by the first phase of a doctoral research project that aimed to develop and validate a bilingual language assessment test for IsiZulu-English-speaking children in grades 1, 2 and 3 with language-based learning disabilities (L-b LDs) in South Africa.

**Objectives:**

Phase 1, systematic literature review, pretesting and formulating of a theoretical framework, with the aim to determine early indicators of L-b LDs; this is important for developing a clinical language test as it determines its constructs.

**Method:**

Thematic analysis was used to develop the models.

**Results:**

This article reviews the literature on indicators and definitions of L-b LD, introduces models that were developed in the study to conceptualise L-b LD and discusses implications for language test development.

**Conclusion:**

The models provided in this article conceptualise L-b LD and identify its early indicators. The application of these models in both educational and clinical settings is proposed for differentiation of L-b LD.

## Introduction

IsiZulu is the first language to 78% of the population in the KwaZulu-Natal province, but English is most often used as a language of learning and teaching (LOLT; Department of Basic Education, 2012; Statistics South Africa, 2018). This puts English second language learners at a higher risk for learning disabilities (Burr, Hass, & Ferreir, 2015). When an isiZulu–English-speaking learner faces difficulties in the classroom, it is critical to distinguish which type of language impairment they could have. Firstly, it could be because of language differences, which is related to differences expressed in semantic structure, speech sound production, vocabulary and pragmatics between the first and second languages (De Lamo White & Jin, 2011). Secondly, it could be a primary language impairment, which is a significant deficit in language ability, affecting the first language, which cannot be attributed to hearing loss, low non-verbal intelligence or neurological damage (Leornard, 2014). Thirdly, it could be a specific learning disability (SLD), referred to here as language-based learning disability (L-b LD), which is a group of disorders in listening, speaking, reading, writing and mathematics (American Speech-Language Hearing Association, 2015).

This could be one of the scenarios where the learner is referred to speech language therapy (SLT) for diagnostic differentiation. Thus, it is critical to have a strong foundation to evaluate the factors that contribute to poor academic performance, particularly in literacy. Considering this challenge, the relevance of speech and language assessment is underestimated within the education sector in South Africa because of neglect of the connection between language processing and learning. Clinical and non-clinical indicators have been proven to predict the possibility of L-b LD (Mann, McCartney, & Park, 2007; Mazzocco & Thompson, 2005; Murray & Wren, 2003). Early prediction has benefits of early intervention that includes timeous referral for SLT, remediation of weak skills and reducing the effects of the impairment on the child, family and education system (Miller, Vaughn, & Freund, 2015).

The significance of this article is based on the unknown prevalence of L-b LD in South Africa, with the recent results released by Progress in International Reading Literacy Study (PIRLS) indicating that 78% of South African grade 4 learners could not read for meaning (Mullis & Martin, 2017). The PIRLS suggests that the reasons for poor reading are not unique to South Africa, although some extrinsic or contextual factors, such as bullying and teacher training, have also contributed to poor learner perfomance (Mullis & Martin, 2017). With literacy being part of our role as speech and language therapists, it is our mandate to offer solutions to assist learners with L-b LD as it manifests in multiple domains of academic functioning, and primarily in those of literacy, such as vocabulary acquisition, reading and writing (American Speech-Language Hearing Association, 2015). Moreover, identification of early indicators of L-b LD relevant to the South African context contributes to a greater task of developing assessment tools. Thus, this article highlights the perspectives speech and language therapists should consider in assessing L-d LD.

## Global perspectives on learning disability

Learning disability has been historically defined differently across the globe over the century. The fifth edition of the *Diagnostic and Statistical Manual of Mental Disorders* states that SLD is ‘history or current presentation of persistent difficulties in the acquisition of reading, writing, arithmetic or mathematical reasoning skills during the formal years of school’ (p. 1) (American Psychiatry Association, 2013). The clear identification of the academic manifestations in the American Psychiatry Association’s definition indicates that the identification of SLDs is based on academic skills, despite the nature of a presumed underlying and unspecified central nervous system disorder (Montague & Cavandish, 2013). Moreover, the key academic manifestations of SLDs are associated with a conceptualisation of a learning disability that has to do with a language processing disorder (Uys, Van Der Walt, Van Der Berg, & Botha, 2007).

Because L-b LD is viewed as a brain-based difficulty, it is an intrinsic condition with specific causative factors (National Center for Learning Disabilities, 2013). Examples of causative factors include perceptual disabilities, brain injury, minimal brain dysfunctions, dyslexia and developmental aphasia (American Speech-Language Hearing Association, 2015). Some definitions also incorporate the contribution of using more than one language in the home or school, with multilingualism being incorporated as one of the extrinsic (contextual) causative factors (American Speech-Language Hearing Association, 2015).

## Defining learning disability in South Africa

Defining learning disabilities in South Africa is multidimensional, as it incorporates intrinsic and extrinsic barriers to learning. The intrinsic barriers are generally developmental, and incorporate a wide range of disabilities, including physical, visual, hearing and psychological conditions (Department of Education, 2005). Academic difficulties experienced because of various factors, including physical, environmental and contextual disadvantages, are referred to as extrinsic barriers to learning (Department of Education, 2001). The terminology of barriers reflects sensitivity towards people with disabilities, promotes inclusivity and acknowledges that L-b LD is mainly recognised in academic settings. Meanwhile, the term ‘barriers’ is extremely broad and sometimes confusing among professionals, as the term does not distinguish between L-b LD and other disabilities associated with social, cognitive or contextual factors related to education and policies (Scanlon, 2013). Generally, it is believed that *learning difficulties* arise from extrinsic factors (contextual) while *learning disabilities* are a result of intrinsic factors (specific to each person) and may persist in the presence of ideal learning conditions and support (Zuma & Dempster, 2008).

South African studies further suggest that intrinsic and extrinsic factors interplay in the manifestation of SLDs (Nel & Grosser, 2016). There is strong evidence that cognitive and intellectual disabilities also form part of the group of barriers that can affect learning, reasoning, problem solving and memory negatively (Jooste & Jooste, 2011). There is an equally strong sense that socioeconomic factors, such as poverty and nutritional issues, affect the physical and socioemotional well-being of a child (Geldenhuys & Weavers, 2013). When the support structures are not functioning adequately between the educational and health systems in terms of detecting learning disabilities, there are also discrepancies in assessing and rehabilitating learners with L-b LD (Nel & Grosser, 2016). At policy level, there are guidelines for screening, identification, assessment and support for learners with L-b LD (Department of Education, 2008). However, in practice, there is still poor integration of services between schools and therapeutic services, as teachers and allied health workers struggle to translate policies into practice, specifically in the fluctuating public sector environment (Engelbrecht, Nel, Nel, & Tlale, 2015). This suggests continuous work into practices and models of identification and intervention for L-b LD.

## Theoretical model of language-based learning disabilities for South Africa

A number of explanatory models have been proposed to guide the determination of learning disabilities. These include comprehensive frameworks that are conventional, such as the cognitive processing, and alternative models that seek to extend beyond low achievement to ‘responsiveness to intervention’ and dynamic assessment (Fuchs, Compton, Fuchs, Bouton, & Caffrey, 2011; Johnson, Mellard, & Buryd, 2005). Therefore, key components have been identified to guide models and provide a comprehensive approach to L-b LD. Scruggs (2003) emphasised that a model should preserve a concept of L-b LD that includes various aspects beyond low achievement; ensuring the identification of learners with L-b LD reliably for differentiation; acknowledging its multifaceted nature, not only reading difficulties, and its persistence across the life span; as well as establishing the technical adequacy of assessment measures across the school.

**FIGURE 1:**
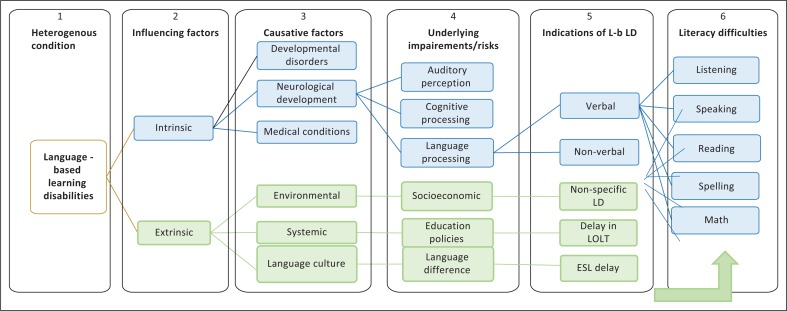
Model describing language-based learning disability for South African context.

Since L-b LD has underlying neurological processes, a model should apply to all language and cultural groups showing consistency in both the concepts and operational definitions of L-b LD settings (Sideridis, 2007). Thus, Figure 1 illustrates the composite models of definitions of L-b LD developed for this research project as compiled by the authors. Defining and conceptualising L-b LD in South Africa is complex; thus, the model acknowledges the broad nature of learning disabilities and various factors that directly and indirectly contribute to it, supported by the global literature (Anthony, Anthony, & Dunkelberger, 2011).

The terms SLD, ‘language learning disability’ and ‘dyslexia’ are often used interchangeably by professionals and parents alike. The term L-b LD instead of the more generic ‘SLD’ is used in this article for accuracy and clarity. In Box 1, the model adopts L-b LD as a merging concept from various definitions of learning disability (National Joint Committee on Learning Disabilities, 2007). The emphasis on ‘language-based’ highlights specific verbal rather than non-verbal factors that influence learning disability (Fuchs, Deschler, & Reshly, 2004). L-b LD is also preferred because of the relationship between spoken and written language, as many learners with reading problems also have spoken language problems (Roseberry-McKibbin, 2008).

Box 2 in the model shows that, broadly speaking, there are intrinsic and extrinsic factors that predispose to L-b LD. The intrinsic factors, as shown in Box 3, mainly relate to neurological development disorders and medical conditions that predispose to L-b LD. The neurological development disorders are linked to possible underlying impairments in auditory perception, cognitive skills or language processing skills, as indicated in Box 4 (Mazzocco & Thompson, 2005; Swanson & Alexander, 1997). Box 5 shows that impairments in both verbal and non-verbal skills interact to present L-b LD reflected in literacy difficulties in learning outcomes of listening, speaking, reading, spelling and numeracy shown in Box 6 (National Center for Learning Disabilities, 2013).

Regarding extrinsic factors, in Figure 1, causal factors are because of environmental, systemic or contextual dynamics. Of these, language culture is critical to L-b LD although other factors may affect its severity (Sideridis, 2007). Language culture is a concept based on the social identity theory, which suggests that an individual’s social identity represents an amalgamation of cultures across boundaries that fuse together to create one’s own culture and combinations that is unique to each individual. Viewing culture through this lens prevents categorising individuals by race, tribe or religion because individuals may not belong to the same culture despite being a member of the same ‘culture’ through family or country (Straub, Loch, Evaristo, Karahama, & Srite, 2003). In the model, language culture can be formed through lifestyle, use of English as a second language, exposure to multiple languages in different contexts, language support and school attended. Language factors such as language difference, incompetency in different languages used in the context of multilingualism and use of English as a second language at school are not impairments but risk factors that may be indicative of L-b LD for South African children and contribute to literacy difficulties in listening, speaking, reading, spelling and mathematics (National Joint Committee on Learning Disabilities, 2007).

The definition of L-b LD proposed by the model is: L-b LDs are difficulties in listening, speaking, reading, spelling or mathematics that manifest because of limitations in auditory perception, cognitive and language processing skills that can be caused by various intrinsic factors, including developmental, neurological or medical conditions, and influenced by extrinsic factors, such as environmental, systemic, language or cultural factors.

## Theoretical approach to early prediction

The systems approach views processes as parts of an overall system, all of which need to function for the system to work well (Friedman & Allen, 2014). Many systems impact a learner’s developmental outcomes but this study focused on six that influence the proximal system of the learner with L-b LD. The adoption of the systems approach was based on the concept of the adjusted Process–Person–Context–Time model, which states that different systems are the contexts of an individual’s development (Bronfenbrenner & Morris, 2007).

**FIGURE 2:**
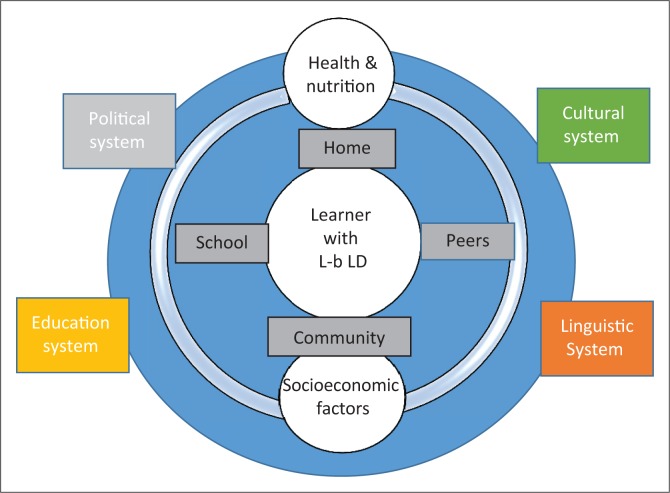
The system of language-based learning difficulties.

The interactions between different systems affect student achievement, staff training and development programmes (Furst-Bowe, 2011). Relating the systems approach to a learner with L-b LD, the parameters in a learner’s home, community or school environments may invite, permit or inhibit engagements in sustained, progressively complex interaction with the immediate environment, forming microsystems (Bronfenbrenner, 1994). Figure 2 also provides a schematic view of some of the possible mesosystems that interact with macro systems that should be considered for clinical language assessment. Assessment should incorporate foundational knowledge in physiological (age, health and nutrition), educational (grade and language difficulty status), linguistic (languages spoken at home and language experience in English as LOLT), cultural (beliefs and values), political (school attended) and social (group culture, norms and practices) systems (Brea-Spahn, 2014).

The systems approach applies in the South African context as barriers to education can be found in the school, individual learner and home levels (Navsaria, Pascoe, & Kathard, 2011). Other systemic factors in politics, health, education and economics are acknowledged as likely to exacerbate an existing L-b LD (Mann et al., 2007). Thus, speech and language therapists must consider the whole system affecting the learner with L-b LD and attend to intrinsic and extrinsic indicators for both assessment and intervention.

## Early predictors of language-based learning disabilities in South Africa

**FIGURE 3:**
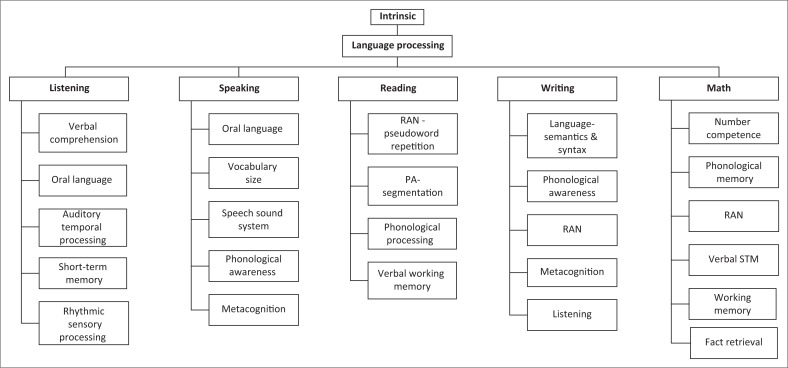
Intrinsic indicators of language-based learning disabilities.

Generally, developmental delays in speech and language, cognitive processing skills, perception, reasoning, social interaction, motor coordination and other developmental areas relevant to meeting educational goals are indicative of some form of learning disability (Baddely, Gathercole, & Papagno, 1998; Frijters et al., 2011). These indicators may occur concomitantly with problems in self-regulation, attention or social interaction (Tranter & Kerr, 2016). Although various systems contribute to L-b LD, research on early intrinsic predictors has mainly focused on the neurological and physiological factors. Using thematic analysis where patterned meanings across journal articles were analysed, a flow diagram was created for this article to summarise the intrinsic L-b LD indicators that were evaluated in our study. Figure 3 focuses on language processing tasks, bearing in mind that they indirectly assess underlying cognitive processing functions (De Lamo White & Jin, 2011). These subskills inform the possibility or risk of L-b LD, referred to in the model as intrinsic early indicators. To align with global definitions of L-b LD and South Africa’s curriculum learning outcomes, the language processing indicators were grouped into five sections, as also indicated in Box 6 of Figure 1.

**FIGURE 4:**
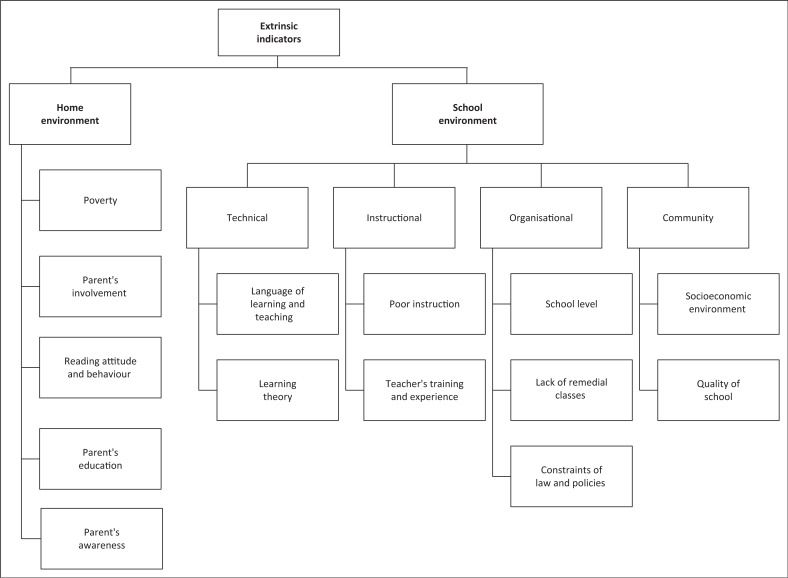
Extrinsic indicators of language-based learning disabilities.

Since these intrinsic indicators are based on underlying neurological processes, they are generally assumed to be universal and cross-lingual (Frijters et al., 2011). Extrinsic factors in the home and school contexts have been established as predictive for L-b LD while socioeconomic, political, educational and cultural systems are indicative of the severity of the learning disability as well as the success of an intervention or performance (Mann et al., 2007). Figure 4 details the extrinsic factors identified in the literature review as relevant to the South African context. In Figure 4, poor instruction, teacher’s negligence in the class, lack of remedial classes and the overall school environment have a major contribution to early prediction of L-b LD and can perpetuate L-b LD (Kavita, Shamilla, & Darshan, 2012).

The recent PIRLS supported the effect of various school-related factors as impacting English second language learners as it found teachers to be insufficiently equipped to teach ESL learners. A minority possessed graduate and postgraduate training proving that teacher training and experience has a contribution to L-b LD identification (Willenberg, 2018). Additionally, bullying, a community-level contribution, negatively affected 42% of learners in South Africa (Willenberg, 2018). In some South African homes, parental awareness, family literacy levels and reading behaviour were found to be significant contributing factors to reading difficulties (Willenberg, 2018). Poverty affects at least 55.5% of South Africans and remains the driving force for parental behaviour such as low parental involvement in school, poor access to books, negative reading behaviour and parent anxiety once the child is diagnosed with L-b LD (Mullis & Martin, 2017; Statistics South Africa, 2017). The challenge is not only on acknowledgment of these factors in L-b LD assessments but in the application of remedial actions that affect the home and school levels as well.

### Ethical considerations

This research was approved by the Biomedical Research Committee of the University of KwaZulu-Natal in 2014 as well as the Department of Education in KwaZulu-Natal province.

## Discussion

The purpose of language assessment in the determination of L-b LD is to differentiate between L-b LD and other language disorders in order to inform and support parents and educators. Clinical assessment aims to determine the core indicators of L-b LD including those that are abstract, uncommon and context specific. L-b LD assessment, based on the models presented, should account for all intrinsic and extrinsic indicators, as well as consider carefully the impact of the home and learning environments.

An assessment approach that combines qualitative and quantitative methods and takes a holistic approach to analysis and intervention is encouraged. Heavily criticised norm referenced measures assume that children are exposed to the same concepts, vocabulary and life experiences (Laing & Kahmi, 2003). Hence, application of sociocultural assessment principles is highlighted here with the models identifying parameters for assessing different dimensions of a learner. This approach allows for parent and teacher involvement in assessment, and considers age-related maturational changes, the influence of language, culture, and school experience on test performance and learning outcomes in intervention. A recommendation from this article is use of grade levels instead of age as an alternative to interpret language test results as studies in sub-Saharan Africa found less robust association of chronological age with test performance (Kar, Rao, & Chandramouli, 2008; Nampijja et al., 2010).

This article also suggests that universal early predictors of L-b LD need to be confirmed for South African languages through research and applied appropriately for isiZulu language, not just because it is ethnographically transparent but also because of different language cultures (Zuma & Dempster, 2008). Some historical differences between South African English and isiZulu cultures involving children are still relevant in the present day. These influence how people speak and interpret what happens in their lives including diagnosis with illness and disabilities. A mixture of Christian and ancestral belief, use of traditional healing, Zulu parenting styles, family concepts and Zulu folklore are some examples of persistent cultural differences that contribute to different language cultures (Singh & Rampesad, 2010). Using the presented models is not just for acknowledgement of cultural factors in assessment but focussed on using this diversity for impact in intervention. Speech language therapies should explore use of current technology and social media platforms such as Instagram and WhatsApp groups to inform teachers, involve parents and support therapy.

Additionally, this article questions the relevance of some indicators to Nguni languages. For instance, rapid automated naming and forward digit span are well recognised as predictors of L-b LD; however, poor performance on the digit span test was observed in Gambia and linked to linguistic bias for Wolof-speaking learners (Jukes & Grigorenko, 2010). Secondly, urban placement of learners may be an advantage as better performance of urban learners from sub-Saharan Africa in tasks such as digit span was found to be because of adaptation and fostering of cognitive skills (Jukes & Grigorenko, 2012). Thirdly, the nature of the assessment task may not be linguistically appropriate. In a South African study, typically developing Afrikaans-speaking grade R learners obtained an average score of 52% while the score for monolingual English-speaking children was 55% on a digit span test (Gagiano & Southwood, 2015). Notably, both groups performed much better in sentence repetition tasks, achieving an average of 73% in Afrikaans speakers and 86% for English speakers. Additionally, the use of English items and academic-based concepts of letters and numbers was noted to be a limitation for a working memory test involving isiZulu primary school children (Mazibuko, 2018). Therefore, what appears to be minor language differences may yield to significant statistical differences. Hence, there should be emphasis on verifying the applicability of universal early predictors and the implications for each language.

## Conclusion

The prevalence of L-b LD is unclear in South Africa because of the lack of nationally accepted definitions across the health and education spectrum, and the interplay between intrinsic and extrinsic factors that result in L-b LD. Thus, it is essential to define the term ‘learning disability’ clearly, conceptually and contextually. This article presented three models to conceptualise L-b LD and identify its early indicators. The application of these models in both educational and clinical settings is proposed for differentiation of L-b LD. These models have been utilised in the blueprint of a bilingual language test for isiZulu–English grade 1, 2, and 3 learners. That test sought to differentiate between L-b LD and normal language behaviour of multilingual learners or process of ESL language development, taking note of linguistic, cultural and systemic contexts. This article contributes to a discussion of fair clinical language assessment practices and use of innovative methods and tools designed for South African population. It encourages debate regarding evaluating the language and literacy concepts for different language groups in South Africa.
